# *Taming, Domestication* and *Exaptation*: Trajectories of Transposable Elements in Genomes

**DOI:** 10.3390/cells10123590

**Published:** 2021-12-20

**Authors:** Pierre Capy

**Affiliations:** Laboratoire Evolution, Génomes, Comportement, Ecologie CNRS, IRD, Université Paris-Saclay, 1 Avenue de la Terrasse, CEDEX, 91198 Gif-sur-Yvette, France; pierre.capy@universite-paris-saclay.fr

**Keywords:** transposable elements, domestication, exaptation, taming, genome plasticity

## Abstract

During evolution, several types of sequences pass through genomes. Along with mutations and internal genetic tinkering, they are a useful source of genetic variability for adaptation and evolution. Most of these sequences are acquired by horizontal transfers (HT), but some of them may come from the genomes themselves. If they are not lost or eliminated quickly, they can be tamed, domesticated, or even exapted. Each of these processes results from a series of events, depending on the interactions between these sequences and the host genomes, but also on environmental constraints, through their impact on individuals or population fitness. After a brief reminder of the characteristics of each of these states (taming, domestication, exaptation), the evolutionary trajectories of these new or acquired sequences will be presented and discussed, emphasizing that they are not totally independent insofar as the first can constitute a step towards the second, and the second is another step towards the third.

## 1. Introduction

Transposable elements (TE), frequently called “*selfish genes*” [1], “*selfish DNA*” [2], or junk or garbage DNA, according to the categories defined by Graur et al. [3], present several genetic characteristics that allow them to rapidly invade genomes and populations, as well as to sometime to settle there permanently. Generally, after their arrival in a naïve genome and an invasion phase, their overall activity decreases drastically, leading to the maintenance of very few autonomous copies. However, several non-autonomous or dead copies, or even pieces of TE, can be preserved with non-neutral effects on individual fitness, due to their particular insertion site or the acquisition of new characteristics after a more or less lengthy coevolution with genomes.

During this coevolution process between TE and genomes, various interactions and trajectories can lead to the emergence of relatively stable evolutionary states, usually described as taming, domestication, or exaptation. Although these different terms seem to be closely related, they cover different phenomena, as briefly described below.

*Taming*. This interaction tends to rapidly reduce and limit the negative fitness impact of an excessively high transposition rate of a new invading TE on both genome structure and function. This is not an irreversible phenomenon because, sometimes, it must be reset at each generation, especially if it is due to non-transgenerational epigenetic marks. Moreover, in stressful conditions, an element can escape and have an intensive transposition activity. This can be illustrated by the regulation of TE activity, with occasional wake-up and bursts [4,5,6,7,8,9]. In this respect, the epigenetic regulation of TE activity plays an important role, and a few autonomous and silenced copies present in the genome can be reactivated occasionally by biotic, abiotic, genomic, or demographic stress. At the populational level, this is crucial for creating new genetic variability to cope with stress and adapt to new environmental conditions. 

*Domestication*. The general definition of domestication is: a sustainable interaction, maintained over generations, resulting from a hierarchical relationship, based on a directional transformation of one entity by another for its own benefit. This leads to deep modifications of genetic material of the domesticated entities, like acquisition, loss, or transformation of one or several traits. In a genomic context, TEs are the domesticated entities and genomes of the “hierarchical superior”. Moreover, while there is no emergence of a new function, they can have an impact on the genome’s functioning. Indeed, a copy, through its genomic insertion site, can impact individual fitness and rapidly invade and settle in the population if it provides an advantage. In this respect, work based on populational analyses has reported many examples [10,11,12,13].

*Exaptation*. This term, introduced by Gould and Vbra in 1982 [14], refers to the emergence of a new function that enhances the fitness of individuals. More precisely, it (in Table 1 of their publication) suggests two different processes: “*1—character, previously shaped by natural selection for a particular function (an adaptation), is coopted for a new use-cooptation; 2—A character whose origin cannot be ascribed to the direct action of natural selection (a nonaptation), is coopted for a current use-cooptation*”. It is, therefore, a sequential evolution of a trait that was initially shaped (or not) by natural selection to a trait today shaped by natural selection and adapted to a new function. 

Numerous biological examples, at the morphological, physiological, and molecular levels, can illustrate such an evolutionary trajectory, such as the feathers of birds originally “designed” for thermoregulation and today exapted in flight. At the molecular level and, more particularly, in the TE world, several examples will be detailed below. 

During evolution, genetic tinkering is a major source for the emergence of new regulation systems, genome reorganization, and new functions [15,16,17]. Within species, this tinkering may be due to the shuffling and association of different parts of a genome by ectopic recombination, transposition, gene duplication, frameshift mutation, translocation, or, again, autopolyploidy in plants. However, this dynamic can also be fueled by the acquisition of external genetic material, as a result, for example, of interspecific hybridizations or horizontal transfers (HT). Such phenomena are responsible for the emergence of genetic novelties, as, for instance, the acquisition of new genes, paralogs of existing genes, and xenologous gene displacement [18]. In addition, they can occur in distantly related species, from different kingdoms within eukaryotes, or even between prokaryotes and eukaryotes. Many example of adaptive horizontal transfers are reviewed by Crisp et al. [19]. According to these authors, 2% of the foreign genes of primates come from archaea, 25% from bacteria, 57.6% from protists, 5.4% from plants, and 10% from fungi.

Based on all genome analyses during the last decade, it has been evidenced that the exchange of genetic material between closely or distantly related species is probably much more frequent than previously assumed. Concerning TE, HT are possible both after an interspecific hybridization or between distantly related species. Nowadays, such transfers do not appear to be rare evolutionary events, and the number of descriptions or suspicions continues to increase [20,21,22,23]. For instance, in insects, Peccoud et al. [22] found that out of 195 genomes, 4500 HT can be detected. 

More precisely, inter-specific hybridizations occur between closely related species, which can hybridize and are able to produce fertile offspring. In plants, such a phenomenon is frequent and leads to the emergence of allopolyploids [24]. This favors the addition of genetic material in both species and the introduction of new variants, which can become the raw material for new genetic tinkering. In animals, inter-specific hybridization can also be observed between species with sexual reproduction. In such a case, and according to Haldane’s rule, only the homogametic sex is fertile (for instance, XX females in the XY system and ZZ males in the ZW system). The fertile sex can then be backcrossed with individuals (males XY or females ZW) of one of the two parental species, leading to the transfer of genetic material of one species to the other (introgression).

On the other hand, horizontal transfers also occur between distantly related species when no sexual reproduction is possible. They were probably very frequent during the early steps of life [25] and were at the origin of important evolutionary steps, such as the exchanges between prokaryotes and eukaryotes or between bacteria/archaea and extremophilic eukaryotes [26,27]. This also occurs during the endosymbioses of proteobacteria and cyanobacteria, leading to the emergence of mitochondria and chloroplasts [28] or between prokaryotes (see for instance the numerous examples in Escudero et al. [29], or San Millan et al. [30]), where they frequently promote the exchange of resistance to environmental stress via conjugation, transduction, and transformation, whether or not they use TE as vectors [31,32]. 

Since TEs are entities subject to HT between species—the source of genetic variability and tinkering within the genome—it is interesting to detail their dynamics through the evolutionary “dialogue” between them and their genomic ecosystems, after their arrival in a naïve genome. 

## 2. Short-Term Co-Evolution of Transposable Elements and Genomes: Taming

While in prokaryotes, the HT mechanisms are known and responsible for rapid diffusion of resistance to environmental stresses [30], the transfer mechanisms remain unknown in eukaryotes, and several scenarios have been proposed [21,33,34]. However, it is likely that the arrival of a new TE in a eukaryotic genome probably occurs in most cases by horizontal transfer [21,35]. At this point in the TE life cycle, there is only one copy in a single individual. Therefore, the probability of losing this copy through genetic drift is very high. To maintain it and allow genome and populational invasion, the impact on fitness must be positive and very high or more likely, TEs have to adopt a parasitic strategy, i.e., a low phenotypic effect, with a relatively high transposition rate [36]. In addition, it seems that several TE, among which some members of the Tc1-mariner superfamily, such as Bari1, Bari3, and Sleeping Beauty would facilitate their genomic diffusion after a horizontal transfer, might have evolved as “blurry promoters” [37,38].

After this more or less lengthy invasion phase, a plateau is reached, during which the number of copies is stabilized. Few copies of this element will then remain autonomous, while the others will become non-autonomous but *trans*-mobilizable, with the remaining copies degenerating. In this context, it is interesting to observe that a competition can take place between the different types of copies from the same family (between autonomous vs. non-autonomous but *trans*-mobilizable copies), leading to a dynamic similar to that described by Lokta [39] and Volterra [40] for the prey-predator relationship in population biology [41,42]. 

This basic TE life cycle can be viewed as a parasitic strategy in the invaded genome. However, the golden rule of many parasitic entities is to be as “silent” as possible. In other words, to be maintained over long evolutionary periods, the TE copy number must be neither too low to avoid elimination by genetic drift or ectopic recombination nor too high to avoid a negative impact on individual fitness. 

In this phase, TE silencing may be promoted by epigenetic regulation. The term “epigenetics” generally refers to several mechanisms, such as cytosine methylation in *Arabidopsis thaliana*, where most copies are methylated and inactivated [43]; small RNA (*si*RNA and *pi*RNA), as described in different tissues in *D. melanogaster* (in germline to control *I* and *P* element transposition [44], testes and ovaries [45,46,47,48]), and somatic and germinal tissues of arthropods [49] (as a stress response in *A. thaliana* [50]), as well as long non-coding RNA in plants with differential expression in tissue and depending on environmental conditions [51]. While the epigenetic regulation seems to be dominant, other mechanisms of TE-silencing can be evoked, such as those involving a self-encoded repressor (such as the internally deleted KP element, derived from the *P* element), [52] or to splicing events, such as for the Bari1 element [53].

One of the evolutionary interests of such silencing is its reversibility. This has two main effects. First, when epigenetic marks are removed, a transposition burst can be observed [54,55] and second, genes located near the TE insertion site can also be reactivated because the methylated area may be larger than the TE itself and can encompass neighboring sequences [56,57,58,59]. Therefore, this type of reversible interaction between TE and genomes can be useful for the genome, insofar as it allows it to maintain a functional “genetic toolbox”, which can be reactivated when necessary to generate new genetic variability and evolve rapidly in a changing environment.

## 3. Long Term Co-Evolution of Transposable Elements and Genomes: Domestication and Exaptation

Two common characteristics are shared by the processes of domestication and exaptation. The first is the “capture” of a copy in a specific genomic location, and the second its maintenance, which can go as far as fixing itself in a population or a species. Regarding the genomic location, this raises the question of the distribution of TE copies in a genome. Is there a random distribution or a patchy distribution with hot insertion regions?

For more than 30 years, it has been observed that TE distribution is patchy [60]. On a coarse scale, this distribution can vary from one chromosome to another, but also within a chromosome, and again among the main TE Classes. For instance, in the human genome, the *Alu* distribution is not similar between chromosomes 21 and 22 [61], and L1 elements are not randomly distributed, although they seem able to target all genomic regions [62]. A similar distribution bias is also observed in *Drosophila* [63], catfish [64], and woodpeckers [65], among others. All these results suggest that even if TEs are potentially capable of jumping everywhere in the genomes, purifying selection against new insertion and ectopic recombination can remove several of them and reshape distribution [66,67]. However, the alternative hypothesis, assuming that TEs insert into peculiar regions, cannot be ruled out.

With the accumulation of complete genome sequences and the new molecular tools recently developed to explore them, it is now clear that this distribution is patchy. In addition to the evolutionary forces previously mentioned, new parameters must be taken into account, such as the status (condensation vs. decondensation) of chromatin [68] or “DNA sequence, chromatin and nuclear context and cellular proteins” because they are also involved in TE integration [69], showing that peculiar genomic territories are more prone to TE insertions than others. 

More precisely, several results show that regions with a specific chromatin structure seem to be more “attractive”, such as the regulatory regions of genes or heterochromatin, whether they are centromeric, telomeric or interspersed in euchromatin [70,71,72,73,74]. Insertions of TEs in gene-rich regions have also been frequently described in numerous species, such as *Drosophila* for retrotransposons [75], for the retroposon *Accord* in 5′ of a gene involved in resistance to insecticides [76,77,78], and, more recently, for diverse TE families, frequently associated with stress-related genes [79]. Similar observations have been reported in mice [80] and wheat [81]. Moreover, the existence of nested accumulation of TEs in euchromatin [82], useful for TE “paleontology” [83], must also be considered. Especially, since they could be at the origin of Pi clusters, involved in regulation of TE activity by small RNA [48,84,85,86,87]. 

Some regions are the main targets of TEs, probably because of their accessibility [88,89,90]. In addition, patchy distribution due to the accessibility effect could be reinforced by the existence of low recombination rates, leading us to consider some of these regions as TE graveyards [91,92,93,94].

Therefore, patchy TE distribution is the result of multiple factors, and two steps must be considered: first, an insertion phase with random or non-random insertions, and second, a differential elimination or maintenance phase, due to selection against deleterious insertions, positive selection on insertion with beneficial host impact and elimination in regions with a high recombination rate. 

In this review, I will differentiate *domestication* and *exaptation*. Can an insertion close to a gene and modifying its expression profile not be considered as an exaptation? Although such insertions have an impact on the host genome, as illustrated by many examples such as Mendel’s wrinkled pea [95], the industrial melanism of *Biston betularia* [96], the resistance to insecticides [77,97,98] or to xenobiotics [78] in *D. melanogaster*. Their frequency may increase in natural populations more or less rapidly, depending on their effect on host fitness [99] and/or the genetic drift, due to the effective population size (*Ne*). Domestication applies to a whole TE copy or a part of it, and frequently a copy is completely domesticated as soon as its mobility and its capacity to encode a functional transposition machinery is lost. Whatever the situation, these copies have an impact on the expression profile of the surrounding genes, but they are not initially the source of new functions or new genes. However, domestication can be a step towards exaptation.

On the other hand, in an exaptation process, all or part of the sequence of a copy is fixed in the population or species. This is the source of new functions and sometimes new genes, which significantly increase host fitness. Such novelties are present in a single species when exaptation is recent or in a group of phylogenetically related species for an older exaptation that occurred before the speciation events. Several examples detailed later will illustrate such a phenomenon, such as the telomeric element in arthropods [100], the vertebrate immune system [101], or placenta development in mammals [102].

*Domestication* and *exaptation* can be detected from analysis of the evolution of polymorphism along the chromosome by the existence of regions with low variability due to the effect of selective sweep or background selection. As recently suggested in very interesting articles [103,104], these phenomena require several successive stages. Here, I would just like to summarize this process and add several considerations.

## 4. How to “Capture” a Transposable Element in a Genomic Position

To “capture” a copy at least two steps must be completed: (i) insertion into a particular region and (ii) its maintenance in the population. The insertion may have a phenotypic and a fitness impact, as soon as the copy is inserted, or this impact may occur during the maintenance phase or later as discussed below. 

The first step, at the genome level, is the insertion of a copy in an area of influence. In the present context, an “area of influence” means a region where an insertion can potentially impact the phenotype of the host. In addition, even if at the beginning this insertion is neutral, after various events, such as genetic tinkering and/or events, due to environmental (including genomic) modifications, it can become positively selected. 

Secondly, this insertion must be maintained and “captured”, that is to say that it must increase within the population up to the fixation. This means that the insertion occurring in a single individual, must invade the population and the species. *A priori*, at this stage, the insertion need not be responsible for a phenotype. However, in this particularly critical period if the initial insertion is selectively neutral or slightly advantageous, the probability of losing it by genetic drift is very high [36]. Therefore, the chance of its maintenance in a population or species will be higher if the host fitness is significantly increased. The greater the fitness effect, the faster the invasion will be. 

During the population invasion, the impact of the insertion may be different from that which will be selected later for a new function. The population is not necessarily totally invaded and a frequency-dependent equilibrium can be established. The important point remains the maintenance of the insertion in the population. Then, due to many factors already mentioned (such as genomic tinkering including recombination, mutation, and environmental or genomic changes, among others) a new function may emerge. Such a process is not driven by need but by chance, even if in some cases primary factors, such as environmental, genomic, and populational stresses, can indirectly enhance the emergence of novelties. 

In general, domesticated TEs are not necessarily immobilized, since this phenomenon may concern their activity which can be modulated according to various factors such as stress. On the other hand, exapted TEs are in most cases immobilized since they are at the origin of new functions based on one of their characteristics. However, a few exceptions exist, such as the telomeric elements in *D. melanogaster*. In this species, the LINE elements, *TART*, *Heta-A*, and *Tahre* are still active and jump exclusively to the telomere to act as a telomere maintaining system and to protect them against the erosion due to successive replications [73,105,106]. Moreover, they could have played an important role in eukaryogenesis [100].

## 5. Immobilization of TEs

An immobilized copy can remain partly active if it provides all or part of the transposition elements for other copies. However, unless natural selection keeps it intact, an insertion usually quickly becomes inactive due to an accumulation of mutations. This immobilization of a copy can be done in different ways, in particular by point mutations, insertions/deletions (indels) and truncation (5′ or 3′ ends) recombination. It then degenerates and can disappear, i.e., the TE sequence is no longer recognizable. 

Recombination will occur between regions similar enough to allow them to hybridize. This recombination will lead to a complete or partial loss of a copy after an unequal crossover between two copies. Deletion or inversion will occur after an ectopic recombination between two copies or between repeated sequences within a single copy, as between the LTRs of retrotransposons. In all cases, the remaining copy is a hybrid of the two original ones. Regarding the LTRs of retrotransposons, these sequences at the ends of the elements are usually about 300–400 bp long, and due to the transposition mechanisms of the elements, they are exactly the same just after the insertion of the copy. So, an ectopic recombination between the two LTRs of a copy leaves a solo-LTR containing the sequences involved in the regulatory activity of the element. Such solo-LTRs have been described in many eukaryotes, including fungi [107], plants [108], and metazoans [109]. Some have an impact on the expression profile of surrounding genes and have been retained by natural selection [11,110].

Another mechanism observed for some retroelements, like retroposons, is a truncation due to their insertion mechanism. For example, the insertion and reverse transcription of LINE occur simultaneously. If the reverse transcriptase stops before it has transcribed the entire sequence, this leads to a 5’ truncation. These inactivated copies are called “Dead On Arrival” [DOA copies—see for example, [111,112]. 

For *Class II* elements, in addition to unequal crossover and ectopic recombination, specific mechanisms must be mentioned because they move by a copy-paste mechanism. To fix a copy in a given genomic position, its excision must be impossible or environmentally counter-selected. So, all mutations affecting the transposase-transposon interaction at the fixed site such as the transposase binding site on the ITRs (Inverted Terminal Repeats) will prevent their excision.

Due to the cellular mechanism, induced by the double strand break leaving after an excision, the abortive gap-repair can be at the origin of an internal deletion in DNA transposons [113,114,115]. Indeed, to repair such a break, the copy present on the sister chromatids will be used as a template. But a stop can occur before the complete repair, followed by a hybridization of the two neo-synthetized strands from short direct repeats (SDRs). These SDRs are short sequences of 5–8 bp and thanks to their small size they can be frequent in TE transposons [113,114,115].

**Figure 1:**
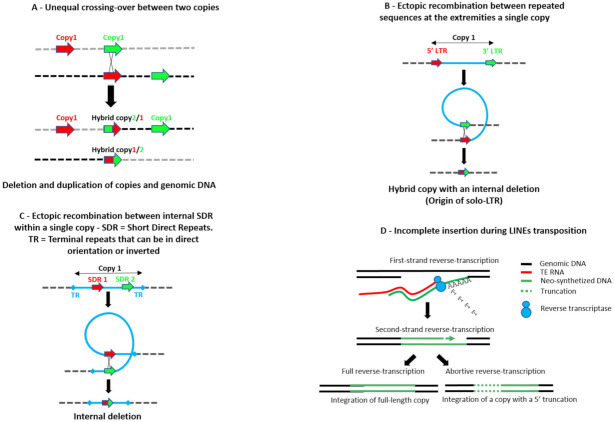

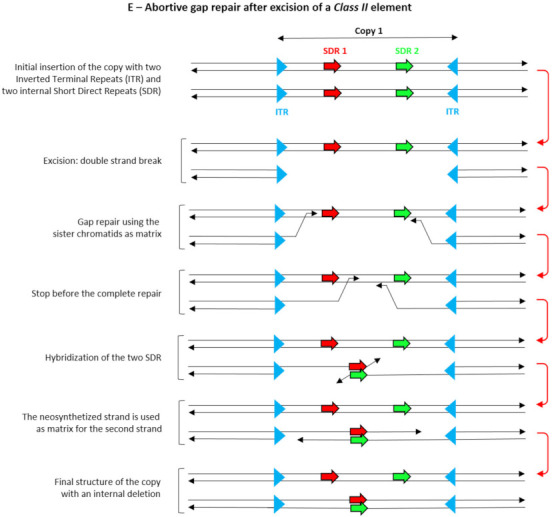
Mechanisms leading to the immobilization of a TE copy insertion, then to its domestication or exaptation. (**A**): Unequal crossover between two copies. This leads to (i) to the deletion of all the sequences between the two copies and (ii) to the duplication of the sequences. In both cases, the new copy (copy 2/1) or the remaining copy (copy 1/2) are hybrid copies due to the crossover between the copies 1 and 2. (**B**,**C**): Ectopic recombination between repeated sequences at the extremities (**B**) or within a single copy (**C**). In both cases, this leads to the emergence of a hybrid copy with an internal deletion. (**D**): Incomplete insertion of retroelements with no LTR. For these elements, the insertion and the reverse transcription (RT) occur at the same time. Frequently, the RT stops before the 5′ end of the element, leading to a 5′ truncation. This is the reason why these new insertions are “dead on arrival”. (**E**): Abortive gap repair. This occurs after the excision of a *Class II* element and has been described in maize and in *Drosophila*. The internal deletion is generated after the detachment of the polymerase and a hybridization of the two neo-synthetized strands thanks to the existence of Short Direct Repeats (SDR) in the TE sequence. This Figure is partly redrawn from Brunet et al. [113]; Levin and Moran [90].

So, the final copy is no longer active and presents an internal deletion between the two SDRs [113,114]. However, such copies can move if both ITRs are preserved and if a source of transposase is present elsewhere in the genome. This can be at the origin of MITEs (Miniature Transposable Elements). All these mechanisms are summarized in Figure 1. 

## 6. Fate of a “Captured” Copy and Emergence of New Functions

Once a copy is immobilized, it usually becomes inactive or dead. There are only few exceptions in which full length elements and their activity are conserved, as in the maintenance of telomere in *Drosophila* species by the LINEs elements *Het-A*, *TART*, *TAHRE*. These active copies jump exclusively to the telomere and prevent their erosion [106,116,117]. Otherwise, even if an immobilized copy can no longer be considered as a TE, some of its characteristics can be recovered occasionally and participate in the emergence of a new function that can be selected in a particular context and increase the average fitness of the population. The distribution of these new functions is generally limited to closely related species or to a single species in the case of a recent exaptation. 

Many examples with a strong selective impact have been reported (see for instance Table 2 of [118]), and new cases are regularly described in eukaryotes, even if the new functions are more or less well established or remain putative. These functions are quite diverse [119,120] and encompass impacts on reproduction [121], the brain [122,123], cell proliferation or death, DNA elimination (*piggyback* [124,125]), vertebrate development [103], diverse CGG-binding protein (TE of the *hAT* family [126]), transcription factors and their binding site (various transposases [127]), new regulatory regions (see for instance *Harbinger* in plants [128]), the emergence of new protein-coding genes via new exon(s), intron(s) with alternative splicing or chromosomal rearrangements [129,130,131], substrate for satellites [132], and small non-coding RNA leading to a kind of immunity against the extension of mobile genetic elements [47,84,133].

At the beginning of the process, the location of the insertion (germinal versus somatic cells) is another crucial point to ensure its inheritance. For unicellular organisms or those in which the germinal line is directly derived from the somatic one, like in plants, this question is not relevant. For other kingdoms, such as the metazoan, such a question is more complicated. Indeed, when an insertion occurs in a somatic line, its inheritance will depend on the stage of formation (early or late) of the germ line during the development. However, exchanges from a somatic to a germinal line have been reported, highlighting that the Weismann barrier is not so impermeable. In addition, several genetic or epigenetic modifications occurring in somatic cells can be transmitted *in-extenso* to germinal cells [134,135,136].

As mentioned above, direct modifications of the germ line are also possible. These can be non-reversible genetic mutations or reversible changes of epigenetic profiles. In the first case, if the mutation occurs late in gametogenesis, it will be present in a single individual of the population, and the probability is very high of losing it [36]. On the other hand, if this mutation occurs early in gametogenesis, it may be present in several individuals, increasing its probability of maintenance in the population. Concerning epigenetic modifications, several individuals in the population can present different epigenetic profiles, and natural selection will retain those (epialleles or combination of epigenetic marks) increasing host fitness. Such modifications can be maintained over a few generations until genetic mutation(s) fix(es) them [137,138,139]. This last scenario can be seen as a “trial and error” process for rapid adaptation with few consequences for the population, since these epigenetics modifications are reversible. 

**Figure 2:**
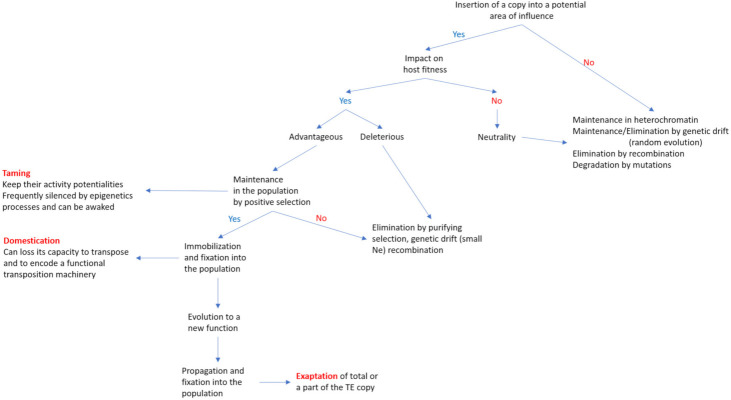
Steps leading to domestication or an exaptation of a TE copy insertion.

**Figure 3:**
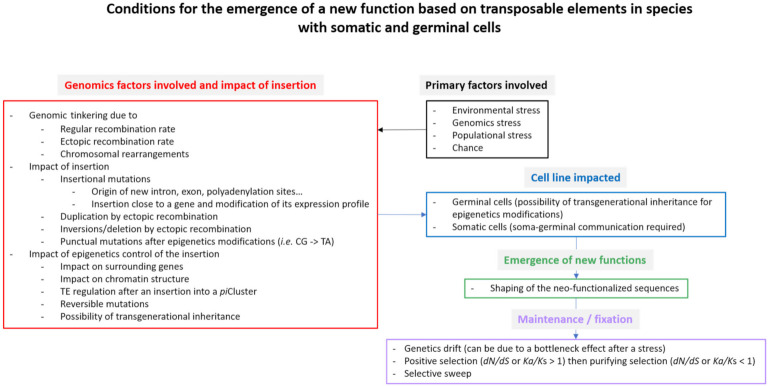
Possible conditions for the emergence of a new function based on a TE copy insertion in species with somatic and germinal cells.

All the events described above in a general context can be applied to TEs. In this case, modifications can be due to new insertions, mutations (punctual mutation, truncation of one or both ends or internal indels) of copies already inserted, involvement in genetic tinkering, epigenetic modifications of copies and of their genomic environment, production of small regulatory RNA (Figure 2 and Figure 3). Several examples of TE impact have already been mentioned at the beginning of the previous paragraph. Otherwise, it has been known for a long while that TE activity and its regulation is different in somatic and germinal lines. More recently, differences were also reported between sexes, even if no general trend has been detected in different taxa. For example, Saint-Léandre et al. [47,48] show that in *D. melanogaster* and its sibling *D. simulans*, TE activity is regulated in ovaries and not always during spermatogenesis. On the other hand, Zamudio and Bourc’his [140] show that in mammalia, inactivation of the factors involved in TE repression leads to male sterility, while in females the same genetic context does not allow the reactivation of TE. Again, Barau et al. [141] show that in some mammals (rodentia and muroidea), the DNA methyltransferase DNMTC3 protects the male germline from retrotransposon activity. All these results clearly evidence that TE activation in both female and male germlines is clearly different.

Moreover, many results can also illustrate the existence of communications between somatic and germinal cells, like the maternal control of TE activity in their descendants. It is now well established that in many animals, mothers can deposit small RNAs in their eggs, which generally prevents TE mobility in their progeny. This is a crucial step, since during early development the zygotic genome is not active, but transcription is based on the maternal products injected into the eggs [142], probably through a gap junction between somatic and germinal cells [143]. For example, in *Drosophila*, the *ZAM* element can be transferred from somatic follicular cells to oocytes using vesicular particles during the vitellogenin transfer [144]. This is probably also true for the *Gypsy* element [145,146]. Since *ZAM*, *Idefix* and *Gypsy* are errantiviruses with a functional *env* gene, such a result is not totally surprising. However, even when the *env* gene is inactivated, such a transfer still occurs [145]. Another example, in *Drosophila*, is provided by the *P* element, recently transferred horizontally into the *D. melanogaster* genome from *D. willistoni*, and which has invaded all the natural populations in a few years [147,148]. The hybrid dysgenesis (sterility of descendants observed when active *P* elements are transmitted from the father and when the mother is devoid of this element) is regulated by small RNA deposit by females in their eggs [149,150,151].

## 7. How to Detect Domesticated or Exapted Copies?

Whole-genome analyses of different species and comparative/functional genomics allow us to follow the emergence and the evolution of domesticated or exapted sequences. In this regard, several approaches can be useful in detecting such sequences. In fact, if a sequence is suspected of having been domesticated or exapted, the questions we can ask are: (i) Is this sequence transcribed or not? (ii) For the coding sequences, what is the ratio dN/dS within species or Ka/Ks between species; (iii) Is the region of its location under a selective sweep?

Regarding the first point, this is not a blocking criterion, since many sequences can have a function without any transcription. For instance, while domesticated/exapted TEs may not be transcribed, they can have a structural function along the chromosome like an insulator with a role as barriers between adjacent genes or to prevent heterochromatin expansion. For example, in *Drosophila* insulators have been described in *Gypsy*, *idefix* and *ZAM* elements [152,153,154]. More generally, TE can be at the origin of promotors, silencers, enhancers and insulator sequences, among others, which are not transcribed. All of these structures can play a major role in regulating gene activity thanks to their conformation and/or the existence of binding sites of several factors [103,155]. On the other hand, even if the sequence is transcribed, this is not a sufficient condition to reject their putative domestication/exaptation because many sequences can be transcribed without a known function via pervasive transcription [156,157,158]. 

The second criterion is much more informative. Indeed, for coding sequences *dN/dS* or *Ka/Ks* ratio gives indications about the type of selection acting on the sequences: a ratio equal to 1 corresponds to selectively neutral sequences, a ratio superior to 1 to sequences under positive selection, and a ratio below 1, a sequence under purifying selection to maintain a function [159]. A *Ka/Ks* < 1 suggests that the new function is already acquired and the purifying selection maintains it by removing all mutations leading to its degradation; while a *Ka/Ks* > 1 means that the sequence is evolving through a new function but that the optimal genetic combination has not yet been reached. For example, the *piggyBac* element found in many eukaryotes has been exapted several times, and these sequences are subject to strong purifying selection [160]. Similarly, in plants several cases of TE exaptation have been reported, such as for the *MUSTANG* gene derived from members of the MULE superfamily [161] or for other TEs [104,119] and in vertebrate for all Host-Transposase Fusions (HTF) tested [127].

The third criterion can also be very informative, but in many cases, there is not enough data to test it. Indeed, such a phenomenon can be observed if the genetic tinkering is recent (at the evolutionary scale), if it strongly increases the fitness of the individuals and if the recombination rate of its genomic location is not too high. The combination of these factors leads to a loss of genetic variability in the genomic region surrounding the loci exapted. This was illustrated in many examples, among which the LINE-like element *Doc* or the LTR retrotransposon *Accord* both involved in insecticide resistance in *Drosophila* [10,77,162] or the TE-derived promoters in humans [163]. Of course, genetic variability will be restored more or less rapidly, according to the substitution rate and recombination rate in this region. 

Finally, another possible clue that can be used when enough copies, both within species and/or from more or less closely related ones, is the TE phylogenies. While these phylogenies are generally incongruent with those of the species, it could be interesting to compare the evolution rate of the different copies. Indeed, even if in these types of constructs are based on a mixture of dead, non-autonomous, and active copies, an indication of a domestication/exaptation can be provided by the existence of long branches, which are the signatures of rapid evolution. Although this hypothesis is not clearly expressed for TE in most of the works, it is nevertheless suspected in a few of them [164,165] and could be combined with the existence of the ratio *Ka/Ks > 1* (if the optimal peak of adaptation is not reached) or *Ka/Ks < 1* (if the peak is already achieved). This can also be related to the rapid evolution of the species if TE domestication is involved in the emergence of one or more genes of evolutionary importance, as suspected in plants [166,167], eukaryotes [168], opisthokonts [169], and vertebrates [127], among others.

## 8. Conclusions and Perspectives 

The relationship between TEs and the genome is not always as simple as one might imagine. On the one hand, when TE activities are modulated by epigenetic marks, the two entities can benefit from such a situation, particularly if epigenetic regulation persists over several generations, due to their inheritance, as described in *Arabidopsis thaliana* [170]. On the other hand, the epigenetic status is reversible, and ETs can be reactivated after a modification of their epigenetic profile, due to, for example, environmental stress. Thus, they become a source of genetic variation, useful for a rapid adaptation of populations but also for themselves, because it allows for the maintenance of active copies, at least, as long as the epigenetic marks are not accompanied by a greater mutability TE immobilizing them. In addition, TEs can participate in their own regulation via the establishment of Pi cluster, genesis of small RNAs, or modification of the chromatin conformation. 

In this context, taming, domestication, and exaptation are the result of different trajectories of co-evolution. While taming must be rapid to avoid a strong impact on population, fitness, domestication, and exaptation take longer, as described throughout this review. 

## 9. Remaining Questions

To be as complete as possible, many questions remain unsolved, such as those mentioned by Jangam and his collaborators [119]. Indeed, although the cases of domestication or exaptation are steadily increasing in the literature, their number remains too low to draw general conclusions. However, from all the analyses available today, it should be noted that:

(i) TEs (or viruses) are more frequently domesticated/exapted than any other type of sequence;
(ii) DNA elements seem to be recruited more frequently than other types of TE (retrotransposons, etc.);
(iii) Some elements exhibit recurrent domestication/exaptation, such as *P* in drosophila [171], *hAT* or *pogo* in vertebrates [169,172], or *piggyback* in a wide variety of organisms [160,173].

If such trends are subsequently confirmed from the analysis of a larger number of genomes, it will be interesting to try to identify factors responsible for these biases. For the moment, only a few clues can be proposed. 

The answer to the first question probably comes from the repetitive nature of TEs, but also from our ability to identify the ancestral states before domestication. Indeed, it is easier to reconstruct the initial structure of the sequences when a large number of copies is available. Such an exercise has already been performed for other purposes. For example, an active copy of *Sleeping Beauty* has been rebuilt from the analysis of a large number of inactive copies in salmonids [174,175]. So, identifying TE-derived genes is probably simpler. Beyond this problem of detection, another question arises. Does domestication/exaptation occur more frequently in some genomes than in others? Is it a matter of genome size, assuming that TEs in larger genomes should be more prone to domestication/exaptation? We have to be careful with this type of argument, since it can lead to circular reasoning because there is a strong positive correlation between genome size and the proportion of TEs hosted [176,177,178,179]. Thus, before drawing any conclusion, TE composition of the genomes must be analyzed in detail, and the frequency of domestication/exaptation must be weighted by the proportion of the genome occupied by each type of TE (at least the relative proportion of DNA vs. RNA elements). 

In the opposition, DNA vs. RNA elements (our second question), other explanations could be related to a less deleterious effect of a DNA element, due to its smaller size, about two to three time shorter than RNA elements. If so, how can we explain that, in some species, such as *Saccharomyces cerevisiae*, only LTR retroelements can be observed (from *Ty1* to *Ty5*; 91)? However, many other reasons can be put forward, such as their different transposition mechanisms (see, for instance, [127]). Nonetheless, the situation is not so clear, since many short sequences present in LTR of retrotransposons are similar to transcription factor (TF) binding sites found in the regulatory regions of genes, particularly of genes induced by stress [180,181,182]. Such an observation raises a new question: are these short sequences an acquisition of TE from the regulatory region of genes or, on the contrary, an acquisition of genes from TE sequences? Today, no argument supports the first hypothesis, while several observations clearly favor the second hypothesis [180,183]. Moreover, the origin of transcription factors is not restricted to LTR retroelements, but can come from all the different types of TE, some of them suggesting that TE could be “hubs” of TF [127,184].
